# Randomized Controlled Trial of Large Language Model–Assisted Diagnostic Accuracy in Nephrology

**DOI:** 10.1016/j.ekir.2026.106673

**Published:** 2026-06-23

**Authors:** Raphaël Bentegeac, Bastien Le Guellec, Mehdi Maanaoui, Erwin Gerard, Philippe Amouyel, Wisit Cheungpasitporn, Nans Florens, Aghiles Hamroun

**Affiliations:** 1 Public Health - Epidemiology Department, Lille University Hospital Center, Lille, France; 2 UMR1167 RID-AGE, Pasteur Institute of Lille, Institut national de la santé et de la recherche médicale (INSERM), Lille University Hospital Center, Lille University, Lille, France; 3 Department of Neuroradiology, Centre Hospitalier Universitaire de Lille, Universite Lille, Lille, France; 4 U1172-LilNCog-Lille Neuroscience & Cognition, Universite Lille, Lille, France; 5 Nephrology Department, Lille University Hospital Center, Lille, France; 6 U1190 Translational Research for Diabetes, Institut national de la santé et de la recherche médicale (INSERM), Institut Pasteur de Lille, Universite Lille, Lille, France; 7 Centre Hospitalier Universitaire de Lille, ULR 2694 - METRICS: Évaluation des technologies de santé et des pratiques médicales, Institut national de la santé et de la recherche médicale (INSERM), Universite Lille, Lille, France; 8 Division of Nephrology and Hypertension, Department of Medicine, Mayo Clinic, Rochester, Minnesota, USA; 9 Nephrology Department, Hopitaux Universitaires de Strasbourg, Strasbourg, France; 10 Institut national de la santé et de la recherche médicale INSERM) UMR_S 1109 Immuno-Rhumatology Laboratory, Faculte de Medecine, Translational Medicine Federation of Strasbourg (FMTS), FHU Target, Universite de Strasbourg, Strasbourg, France; 11 Investigation Network Initiative-Cardiovascular and Renal Clinical Trialists (INI-CRCT), F-CRIN Network, Strasbourg, France

**Keywords:** artificial intelligence, clinical decision support, large language model

## Introduction

Since the public release of ChatGPT in late 2022, large language models have transformed clinical medicine.^1^ Although advanced models now match or exceed trained physicians on nephrology board certification examinations,^2^ a critical limitation of existing research is the reliance on multiple-choice questions.^2^^,^^3^ Evidence that LLM access improves clinician diagnostic accuracy in realistic settings remains limited and mixed, with early randomized trials reporting divergent results.^4^^,^^5^ In nephrology specifically, no randomized study had evaluated artificial intelligence (AI) assistance using complex, multimodal, open-ended vignettes, leaving the clinical relevance of benchmark results uncertain. We hypothesized that a high-reasoning LLM feedback workflow would improve diagnostic accuracy in complex nephrology cases.

Full methods are provided in the Supplementary Methods section.

## Results

Ninety-seven participants completed 556 case evaluations (no AI: n = 44; AI: n = 53). Median age was 38 years (interquartile range [IQR]: 34–45); 75% reported nephrology as their specialty, 69% were nonacademic, and participants represented 9 countries. Baseline characteristics were balanced between arms (Table 1).

**Table 1:** Baseline characteristics of study participants (*N* = 97)

Characteristic	No AI (*n* = 44)^a^	AI (*n* = 53)^a^
Age, yrs	40 (35–45)	36 (33–45)
Gender		
Female	14 (32%)	16 (30%)
Male	30 (68%)	37 (70%)
Country of origin		
France	41 (93%)	46 (87%)
International	3 (6.8%)	7 (13%)
Certification status		
Board-certified physician	41 (93%)	45 (85%)
Resident/intern	3 (6.8%)	8 (15%)
Specialty		
Internal medicine	7 (16%)	8 (15%)
Nephrology	33 (75%)	40 (75%)
Other specialty	4 (9.1%)	5 (9.4%)
Subspecialty		
Clinical nephrology	9 (20%)	8 (15%)
Dialysis	3 (6.8%)	9 (17%)
Functional exploration	0 (0%)	1 (1.9%)
Generalist	19 (43%)	19 (36%)
Pediatric nephrology	1 (2.3%)	1 (1.9%)
Transplantation	1 (2.3%)	2 (3.8%)
Non-nephrologist	11 (25%)	13 (25%)
Experience years	15 (7, 17)	10 (3, 20)
Academic status		
Academic	15 (34%)	15 (28%)
Non-academic	29 (66%)	38 (72%)
Practice structure		
General hospital	17 (39%)	15 (28%)
Nonprofit organization	5 (11%)	2 (3.8%)
Private practice	3 (6.8%)	6 (11%)
University hospital	19 (43%)	30 (57%)
LLM usage frequency		
Never	17 (39%)	21 (40%)
Monthly	10 (23%)	13 (25%)
Weekly	9 (20%)	13 (25%)
Daily	8 (18%)	6 (11%)
AI confidence level	6 (5–7)	6 (5–7)

AI, artificial intelligence; LLM, large language model.

Baseline characteristics are shown for all included participants and by randomized group. Continuous variables are summarized as medians with interquartile ranges, and categorical variables as counts with percentages. Reported *P*-values are descriptive between-group comparisons and were not used to determine covariate adjustment.

**Figure 1:**
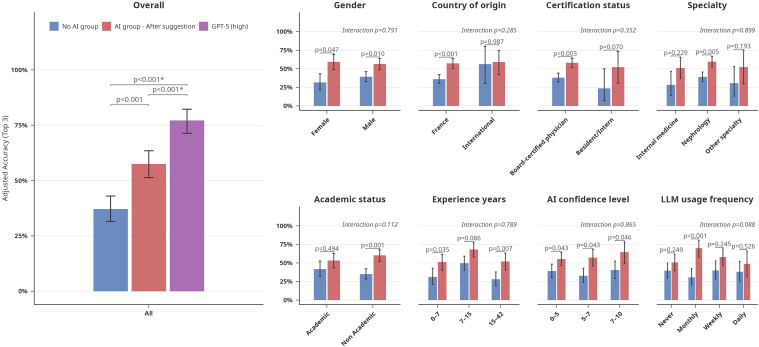
Top-3 diagnostic accuracy by study group and participant subgroup. The left panel shows overall adjusted accuracy in the no-AI group and the AI group after suggestion. Subgroup panels show adjusted accuracy stratified by gender, country of origin, certification status, specialty, academic status, years of clinical experience, AI confidence level, and LLM usage frequency, with *P* values for within-subgroup between-group comparisons and interaction *P* values for each subgroup variable. *P* values comparing GPT-5 alone with clinician groups are descriptive unadjusted 2-sample tests of proportions based on observed top-3 accuracy; they do not account for repeated clinician responses, covariates, or participant/vignette random effects.

Top-3 accuracy was 37.2% (95% confidence interval [CI]: 31.6%–43.0%) in the no AI group versus 57.5% (95% CI: 51.3%–63.5%) in the AI group (adjusted odds ratio [OR]: 2.8, 95% CI: 1.5–5.2; *P* < 0.001; Figure 1, Supplementary Figure S3). Top-1 accuracy followed the same pattern (33.0% vs. 51.5%; adjusted OR: 2.7, 95% CI: 1.5–4.7; *P* < 0.001). Within the AI arm, top-3 accuracy increased from 25.4% before to 57.5% after AI suggestions (adjusted OR: 7.0, 95% CI: 4.4–11.3; *P* < 0.001). Among initially incorrect responses where the AI suggestion was correct, 85 of 178 (47.8%) were corrected after AI exposure; only 1 of 69 initially correct responses became incorrect after viewing an incorrect AI suggestion (1.5%; 95% CI: 0.04%–7.9%; Supplementary Figure S4). Participants revised their top-3 diagnoses in 47.4% of AI-arm responses (95% CI: 41.3%–53.6%). Confidence was slightly higher after AI suggestion than in the no-AI group (median 7 [IQR 6–9] vs. 6 [IQR 3–8]; *P* < 0.001; Supplementary Figure S5). No significant subgroup interactions were detected across gender, specialty, certification status, experience, AI confidence, or LLM usage frequency (all interaction *P* > 0.05; Figure 1, Supplementary Figure S3). Sensitivity analyses restricted to participants completing more than 4 or all 10 vignettes yielded consistent estimates (adjusted OR: 4.3, 95% CI: 2.0–9.3 and 3.9, 95% CI: 1.7–9.1, respectively; Supplementary Table S2). Completion time did not differ between groups (*P* = 0.213).

## Discussion

This randomized trial demonstrates that LLM-supported workflows improve diagnostic accuracy across complex nephrology vignettes, whereas AI-induced errors—initially correct responses that became incorrect after AI exposure—were rare. First, AI-augmented clinicians significantly outperformed unassisted clinicians (57.5% vs. 37.2% top-3 accuracy), but remained substantially below standalone generative pretrained transformer (GPT)-5 performance on the same benchmark (77.1%). This gap underscores that high model accuracy does not translate fully into clinician-AI performance—a pattern consistently observed across clinician-facing AI trials. In the most comparable previous trial, Goh *et al.* found no improvement with LLM access over conventional resources (adjusted difference 2 percentage points, 95% CI: −4 to 8), whereas the standalone model outperformed both groups by 16 percentage points.^4^ Everett *et al.* likewise showed that workflow design matters—collaborative AI workflows improved accuracy over conventional resources (85% and 82% vs. 75%), whereas performance approached but did not surpass AI alone (90%).^6^ Positive result of REFINe (Reasoning Enhancement With Feedback From a Generative AI in Nephrology) likely reflects the complexity of multimodal open-ended vignettes, the absence of competing resources in the control arm, and a frozen suggestion format that may have supported deliberate rather than passive AI use. Whether more naturalistic access, such as a live query interface, alongside other information sources would yield comparable gains remains an open question.

Second, the revision analysis points to where the human-AI interface matters most. Participants revised 47.4% of AI-arm responses, and among the 178 responses initially incorrect when the AI suggestion was correct, nearly half were ultimately corrected, yet half were not. This asymmetry is not a failure of the model; GPT-5 was correct in all 178 cases. It reflects a failure of uptake, consistent with qualitative work showing that physicians tend to filter AI outputs through their existing reasoning rather than accept them uncritically.^7^ The implication is that the user’s ability to recognize and integrate a correct suggestion may be a more important bottleneck than model accuracy per se and that interface design, contextual framing, and structured AI-literacy training may matter as much as model selection. Qazi *et al.* reported a particularly large gain in a low-resource setting after an AI-literacy curriculum (71.4% vs. 42.6%; adjusted difference 27.5 percentage points),^5^ further supporting the idea that user preparation conditions the effectiveness of clinician-AI collaboration.

These findings should be interpreted cautiously—the study was not powered to detect subgroup-level heterogeneity, and a nonsignificant interaction test does not rule out differential effects. A recent dermatology trial reported a similar pattern^8^; however, whether it generalizes requires confirmation in larger studies. If replicated in nephrology, this would have meaningful implementation implications, as AI diagnostic support would not need to be targeted at specific user profiles but could be designed for broad deployment across experience levels.

Fourth, REFINe belongs to a still uncommon class of clinician-facing prospective comparative studies. A recent systematic review of 4609 clinical LLM studies identified only 19 randomized trials among those using real-world data and found that LLMs outperformed human comparators in only 33% of head-to-head comparisons, with outperformance becoming less common as task realism and clinician experience increased.^9^ Against this backdrop, a positive randomized nephrology finding is notable but should be read as part of a still limited and heterogeneous evidence base rather than as definitive proof of generalizable benefit.

Taken together, these findings show that standalone GPT-5 outperformed both clinician groups in this vignette benchmark and that AI assistance brought clinician performance meaningfully closer to that ceiling. Whether this justifies autonomous AI replacement in clinical practice cannot be inferred from structured vignettes alone, which provide curated information around a single diagnosis—conditions differing substantially from real clinical data. LLM output may be more usefully framed as a differential-diagnosis index for clinician verification. The gap between standalone AI and AI-augmented performance (77.1% vs. 57.5%) identifies the human-AI interface as the primary locus for improvement. Several limitations deserve mention. The vignette-based design does not reproduce the fragmentation and noise of real clinical data. Completion was voluntary and uncompensated, and the study evaluated a single proprietary model, limiting generalizability. Subgroup analyses were exploratory and underpowered; null interaction findings should not be interpreted as evidence of uniform benefit. Real-world translation will require prospective evaluation in routine care, with attention to workflow design, over-reliance safeguards, and locally deployable models.

## Disclosure

The authors declared no competing interests.

## Supplementary Material

Supplementary file (PDF) Supplementary Methods. AI Prompting and Model Configuration (PDF). Figure S1. Study design. Figure S2. Benchmarking of candidate models on the full dataset of 245 altered nephrology cases. Figure S3. Subgroup analysis: adjusted odds ratios for AI support on Top-3 diagnostic accuracy. Figure S4. Diagnostic revision patterns before and after AI suggestion (Sankey diagrams). Figure S5. Self-expressed diagnostic confidence across study groups. Table S1. Characteristics of included cases (n = 10). Table S2. Sensitivity analyses by vignette completion level. Checklist 1. TRIPOD-LLM Checklist (PDF). Checklist 2. CONSORT 2025 Checklist (PDF).
