# Supplementary material for: Randomized Controlled Trial of Large Language Model–Assisted Diagnostic Accuracy in Nephrology
Source: Kidney Int Rep. 2026 Jun 23;11(9):106673. doi: 10.1016/j.ekir.2026.106673 (PMC13400205; doi:10.1016/j.ekir.2026.106673)
Supplement: Supplementary file (PDF) — Supplementary Methods. AI Prompting and Model Configuration (PDF). Figure S1. Study design. Figure S2. Benchmarking of candidate models on the full dataset of 245 altered nephrology cases. Figure S3. Subgroup analysis: adjusted odds ratios for AI support on Top-3 diagnostic accuracy. Figure S4. Diagnostic revision patterns before and after AI suggestion (Sankey diagrams). Figure S5. Self-expressed diagnostic confidence across study groups. Table S1. Characteristics of included cases (n = 10). Table S2. Sensitivity analyses by vignette completion level. Checklist 1. TRIPOD-LLM Checklist (PDF). Checklist 2. CONSORT 2025 Checklist (PDF). [file mmc1.pdf]

Randomized Controlled Trial of LLM-Assisted Diagnostic Accuracy in Nephrology

Supplementary Material

Bentegeac R, Le Guellec B, Maanaoui M, Gerard E, Amouyel P, Cheungpasitporn W, Florens N, Hamroun A.

Contents

- Supplementary Methods.
- AI Prompting and Model Configuration.
- Supplementary Table S1. Characteristics of included cases (n=10).
- Supplementary Table S2. Sensitivity analysis by vignette completion levels.
- Supplementary Figure S1. Study design.
- Supplementary Figure S2. Benchmarking of candidate models on the full dataset of 245 altered nephrology cases.
- Supplementary Figure S3. Forest plot of adjusted odds ratios for Top-3 diagnostic accuracy.
- Supplementary Figure S4. Diagnostic revision patterns in the AI group.
- Supplementary Figure S5. Diagnostic confidence by group.
- TRIPOD-LLM Checklist.
- CONSORT 2025 Checklist.

Supplementary Methods

Trial Design and Registration

The Reasoning Enhancement With Feedback From a Generative AI in Nephrology (REFINe) trial was a prospective, randomized, parallel-group, 1:1, superiority, open-label study evaluating the impact of large language model assistance on clinical diagnostic accuracy. The trial was conducted entirely online between November 2025 and March 2026. Reporting followed CONSORT guidelines supplemented by TRIPOD-LLM recommendations. The protocol was approved by the Lille University Hospital Review Board (protocol ID: CHUL-191125), and all participants provided electronic written informed consent. The trial was registered at ClinicalTrials.gov (NCT07352475; first posted November 19, 2025). No important changes to eligibility criteria, outcomes, or analyses were made after commencement; recruitment concluded with 97 rather than the planned 100 participants.

Case Selection and Dataset Preparation

Clinical cases were derived from the *Make Your Diagnosis* series published in *Kidney International* (August 2007 to January 2025). A total of 245 cases were heavily altered by two board-certified nephrologists (N.F. and A.H.) to prevent data leakage and memorization bias, with no sentence or numerical value left unchanged while preserving the underlying clinical logic and final diagnosis. Ten cases were randomly selected from this pool for the trial.

Participants and Randomization

Eligible participants were English- or French-speaking residents or board-certified physicians recruited via professional societies and medical congresses. The primary exclusion criterion was prior exposure to the specific cases used. Upon enrollment, participants provided demographic information including age, gender, country of origin, specialty, years of post-graduation experience, academic status, practice structure, LLM usage frequency, number of AI tools used, and self-reported AI confidence level. Group allocation used the Pocock & Simon minimization algorithm (80% deterministic, 20% random) balancing five characteristics: certification status, specialty, years of experience, LLM usage frequency, and academic status. Randomization was implemented automatically without human intervention; the allocation sequence was concealed from study personnel throughout enrollment.

Trial Procedures

Each participant was assigned the 10 selected vignettes in randomized order via a dedicated online platform with forward-only progression. In the control group, participants reviewed the vignette and provided up to three differential diagnoses (Top-3) and a confidence rating (0–10 scale). In the AI group, participants first provided an initial Top-3 and confidence rating, then were shown pre-generated, frozen GPT-5 suggestions and justifications, and were permitted to revise their diagnoses and confidence a final time. Completion time was automatically recorded. Participants with no completed vignette were excluded; no imputation was required as the platform did not allow partial case submissions.

Trial Outcomes

The primary outcome was Top-3 diagnostic accuracy: presence of at least one correct hypothesis among the three submitted diagnoses per vignette. Adjudication was performed independently by two board-certified nephrologists (N.F. and A.H.), blinded to group assignment; prereconciliation interrater agreement was approximately 97%; disagreements were resolved by consensus. Secondary outcomes included Top-1 accuracy, within-arm accuracy change (before vs. after AI suggestion), self-expressed diagnostic confidence (0–10 scale), AI-induced diagnostic errors (proportion of initially correct Top-1 responses that became incorrect after AI exposure), completion time, and proportion of assigned vignettes completed.

Statistical Analysis

Sample size was determined by simulation-based power analyses using a mixed-effects logistic regression with random intercepts for participant and vignette, assuming 50 participants per arm, 10 vignettes per participant, control-group accuracy 50%, AI-group accuracy 70%, random-effect standard deviations of 0.7, two-sided alpha 0.05, and 500 simulation iterations. A total of 100 participants was estimated to provide greater than 99% power.

The primary analysis used a mixed-effects logistic regression with a fixed effect for study arm and random intercepts for participant and vignette, adjusted for gender, country of origin, specialty, academic status, years of clinical experience, AI confidence, LLM usage frequency, and number of AI tools used. All randomized participants were analyzed in their assigned group. Prespecified subgroup analyses used interaction terms. Post-hoc sensitivity analyses restricted the analysis to participants completing five or more vignettes and to full completers. All tests were two-sided (alpha=0.05). Analyses were performed in R version 4.3.2.

AI Prompting and Model Configuration

Prompting Strategy

All models were evaluated with the same two-stage prompting workflow. In the first stage, the model was instructed to provide exactly three ranked differential diagnoses for each vignette, using case text and associated images when supported. In the second stage, the model expanded those diagnoses into concise, case-specific reasoning with approximate probability estimates and a brief discriminating summary. Prompts were zero-shot; few-shot examples were not used to avoid pattern-matching to cases from the same vignette pool. Prompt refinement was limited to formatting-oriented dry runs and did not use study vignettes, reference diagnoses, or benchmark outcomes.

The two-stage design was intentional: stage 1 ranked diagnoses were fed directly into stage 2, ensuring structured justifications shown to participants were anchored to the same diagnoses scored in the benchmark. The AI output shown to participants therefore consisted of a fixed rank-ordered differential list with concise structured justifications, not a free-form conversation.

First-stage prompt

```
You are an expert clinician. Analyze the following clinical case and provide your
top 3 differential diagnoses.

CRITICAL RULES:
- Provide EXACTLY 3 diagnoses, ordered from most to least likely (one per line)
- Output ONLY the diagnosis names, separated by newlines
- No additional text, explanations, or formatting
- Language: {language}

OUTPUT FORMAT:
Diagnosis 1
Diagnosis 2
Diagnosis 3
```

Second-stage prompt

```
You are a clinical assistant helping physicians with differential diagnoses.

You previously analyzed this clinical case and provided these 3 differential
diagnoses:
{raw_predictions}

Now provide a structured JSON response with concise clinical reasoning for each
diagnosis.

Required JSON format (strict, no additional text):
{
    "intro": "<Brief context - 1-2 sentences, no first-person narration>",
    "specifics": [
        {
            "title": "<Diagnosis name>",
            "description": "<SPECIFIC findings from THIS case supporting this
diagnosis>",
            "probability": <number 0-100>
        },
        ... (repeat for all 3 diagnoses)
    ],
    "remaining_probability": <number 0-100>,
    "outro": "<Key discriminating features from THIS case - no treatment
recommendations>"
}

CRITICAL RULES:
- Be concise and factual; avoid "I will analyze" phrasing
- Reference SPECIFIC findings from THIS case, not generic textbook facts
- Total of all probabilities + remaining_probability should equal 100
- Language: {language}
- Output ONLY valid JSON, no markdown code blocks
```

Benchmarked Models

The benchmarking registry included: gpt-5, gpt-5-mini, o3, gpt-4o, meta-llama/Llama-4-Maverick-17B-128E-Instruct-FP8, google/gemini-2.5-pro, Qwen/Qwen3-VL-235B-A22B-Thinking, deepseek-ai/DeepSeek-V3.2-Exp, alibayram/medgemma:27b, claude-sonnet-4-5-20250929, and magistral-medium-2509.

The model deployed in the intervention arm was gpt-5 with reasoning effort set to high. All benchmark queries and GPT-5 trial outputs were generated in October 2025 and frozen before enrollment. Benchmark inference was parallelized and cached; total runtime approximately 3 hours, total API cost approximately EUR 300.

Cross-model Standardization

All models were benchmarked on the same 245 altered nephrology cases. Most were evaluated in multimodal mode (text and images). deepseek-ai/DeepSeek-V3.2-Exp and alibayram/medgemma:27b were evaluated in text-only mode as image inputs were not supported in those deployment settings. Between-model differences should therefore be interpreted considering both model capability and multimodal availability.

AI Suggestion Example

Vignette (abbreviated): Male born at 36 weeks with diazoxide-responsive congenital hyperinsulinemic hypoglycemia, presenting at age 6 with poor growth, polyuria/polydipsia, and complete proximal tubulopathy with hypercalciuria and medullary nephrocalcinosis.

**Diagnosis**	**Prob.**	**Key supporting findings**
HNF4A-related hyperinsulinism with renal Fanconi syndrome	65%	Diazoxide-responsive neonatal HH + complete Fanconi (pH 7.30, FE HCO3 25%, glycosuria 3+, aminoaciduria, b2-microglobulin >85,000 ng/mL, hypercalciuria, nephrocalcinosis); birthweight 3,100 g at 36 wks.
Nephropathic cystinosis	20%	Explains generalized Fanconi and growth failure. Against: neonatal hyperinsulinism, preserved creatinine at age 6, no extrarenal features.
Dent disease	10%	LMW proteinuria, hypercalciuria, nephrocalcinosis. Against: aminoaciduria, glycosuria 3+, full proximal RTA, neonatal HH.
Other diagnoses	5%	

Key discriminators: diazoxide-responsive neonatal hyperinsulinemia combined with complete Fanconi syndrome most strongly supports HNF4A-related disease.

Supplementary Table S1

Supplementary Table S1. *Baseline characteristics by vignette completion level.*

	Age group	Gender	Presenting syndrome	Nephrology domain	Key modality provided	Broad diagnosis category
1	Pediatric	Male	Fanconi syndrome / proximal RTA	Tubular, interstitial, and cystic disorders	None	Genetic tubulopathy
2	Adult	Female	Acute kidney injury after antibiotics	Acute kidney injury and intensive care	Pathology (kidney biopsy)	Drug-induced acute kidney injury
3	Adult	Female	Nephrotic-range proteinuria (SLE)	Glomerular and vascular diseases	Pathology (kidney biopsy)	Immune-mediated glomerulonephritis
4	Adult	Male	Nephrotic syndrome with dyslipidemia	Glomerular and vascular diseases	Pathology + clinical photo	Inherited metabolic/lipid-associated glomerular disease
5	Adult	Male	Nephrotic syndrome in STI/HIV context	Glomerular and vascular diseases	Pathology (kidney biopsy)	Infection-associated glomerular disease
6	Adult	Female	Severe high anion-gap metabolic acidosis	Electrolyte and acid-base disorders	Urine microscopy	Toxic ingestion / metabolic acidosis
7	Adult	Male	Allograft dysfunction (rising creatinine)	Kidney transplantation	Pathology (allograft biopsy)	Transplant-associated viral nephropathy
8	Adult	Male	Chronic kidney disease with tubular proteinuria	Tubular, interstitial, and cystic disorders	Pathology (kidney biopsy)	Tubulointerstitial kidney disease
9	Adult	Male	Nephrotic syndrome / heavy proteinuria	Glomerular and vascular diseases	Pathology (kidney biopsy)	Nodular glomerulosclerosis-like glomerular disease
10	Adult	Male	Hypokalemic, hypochloremic metabolic alkalosis	Electrolyte and acid-base disorders	None	Electrolyte/acid-base disorder

Broad diagnosis categories are intentionally non-specific to reduce identifiability and leakage concerns. Cases are listed with their broad diagnosis category, nephrology domain, presenting syndrome, and the main diagnostic materials available to participants. Diagnostic materials could include clinical history, laboratory data, pathology images, urine microscopy, or other visual attachments. This table summarizes the 10 altered *Kidney International* vignettes randomly selected for the randomized trial.

Supplementary Tables S2

Supplementary Table S2. *Sensitivity analyses by vignette completion level.*

Sample	N (AI / No AI)	No AI – Top-3 accuracy [95% CI]	AI – Top-3 accuracy [95% CI]	Adjusted OR [95% CI]	p-value
Any completion (≥1 vignette)	97 (53 / 44)	37.2% [31.6%, 43.0%]	57.5% [51.3%, 63.5%]	2.82 [1.54, 5.16]	<0.001
Half completion (≥ 5 vignettes)	52 (23 / 29)	37.4% [31.5%, 43.6%]	60.6% [53.5%, 67.4%]	4.30 [1.99, 9.28]	<0.001
Full completion (10 vignettes)	35 (15 / 20)	41.0% [34.1%, 48.2%]	60.7% [52.4%, 68.5%]	3.94 [1.70, 9.13]	0.001

Adjusted ORs from mixed-effects logistic regression with random intercepts for participant and vignette, adjusting for prespecified baseline covariates.

Supplementary Figures

Supplementary Figure S1. *Study design.*

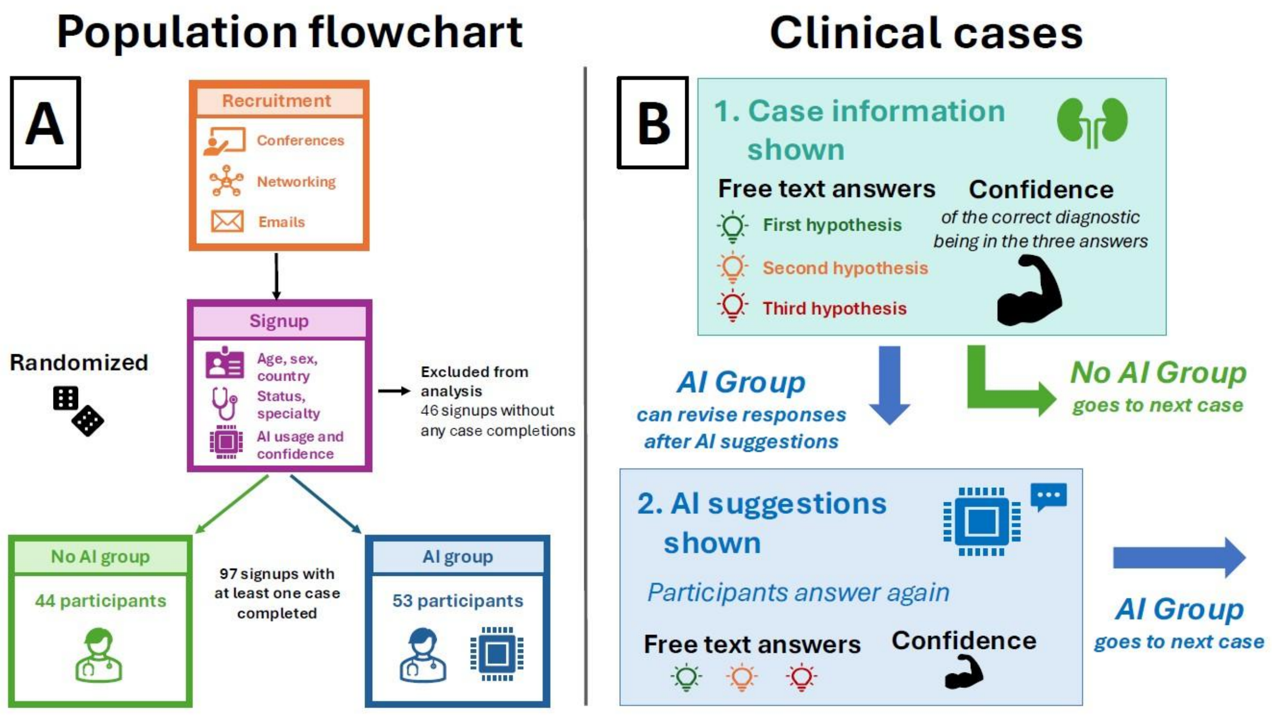

Panel A shows the population flowchart, including recruitment pathways, signup information collected, exclusion criteria, and final group allocation (No AI group: n=44; AI group: n=53). Panel B illustrates the clinical case procedure: participants first provided up to three differential diagnoses and a confidence rating; in the AI group, pre-generated GPT-5 suggestions were then displayed, after which participants could revise their responses before proceeding to the next case.

Supplementary Figure S2. *Benchmarking of candidate models on the full dataset of 245 altered nephrology cases.*

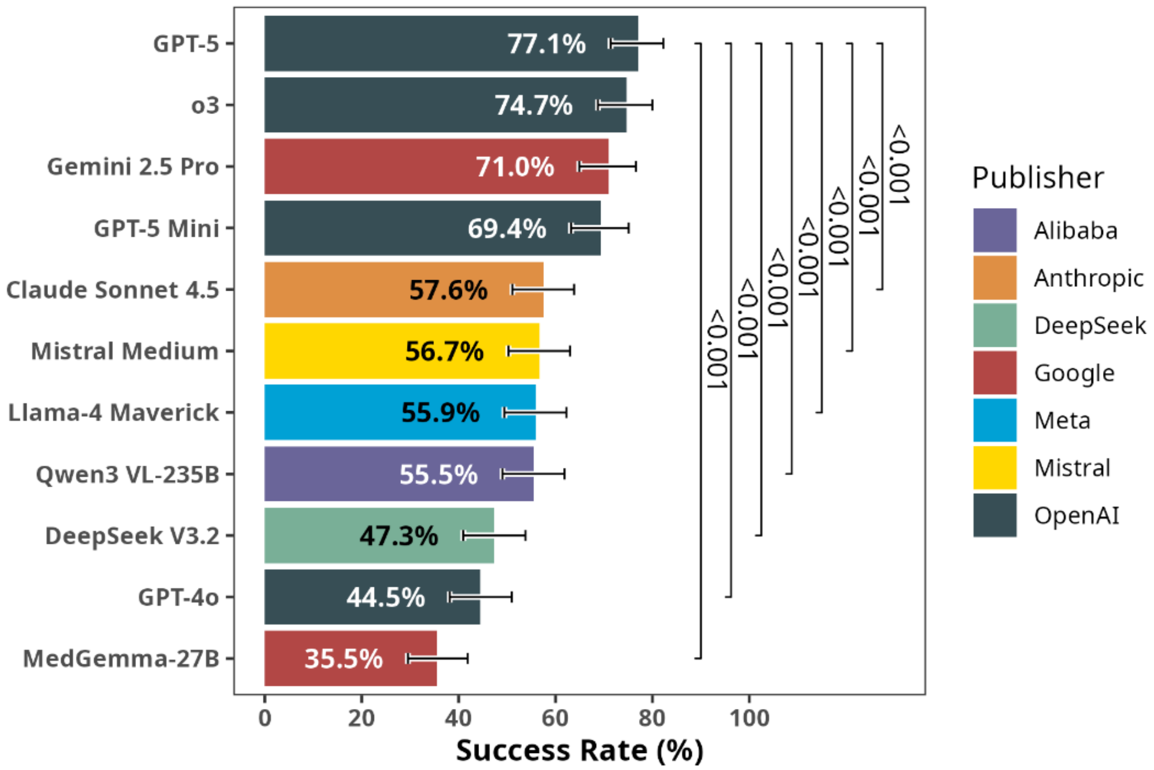

Bars show the raw Top-3 diagnostic success rate for each evaluated model across the full dataset of 245 altered nephrology cases, with error bars indicating exact binomial 95% confidence intervals. Percentages are unadjusted observed success rates. Vertical brackets indicate pairwise comparisons with GPT-5, which was the best-performing model in this benchmark and was therefore selected for the randomized trial. P values are shown for these pairwise comparisons. Colors indicate model publisher.

Supplementary Figure S3. *Subgroup analysis: adjusted odds ratios for AI support on Top-3 diagnostic accuracy.*

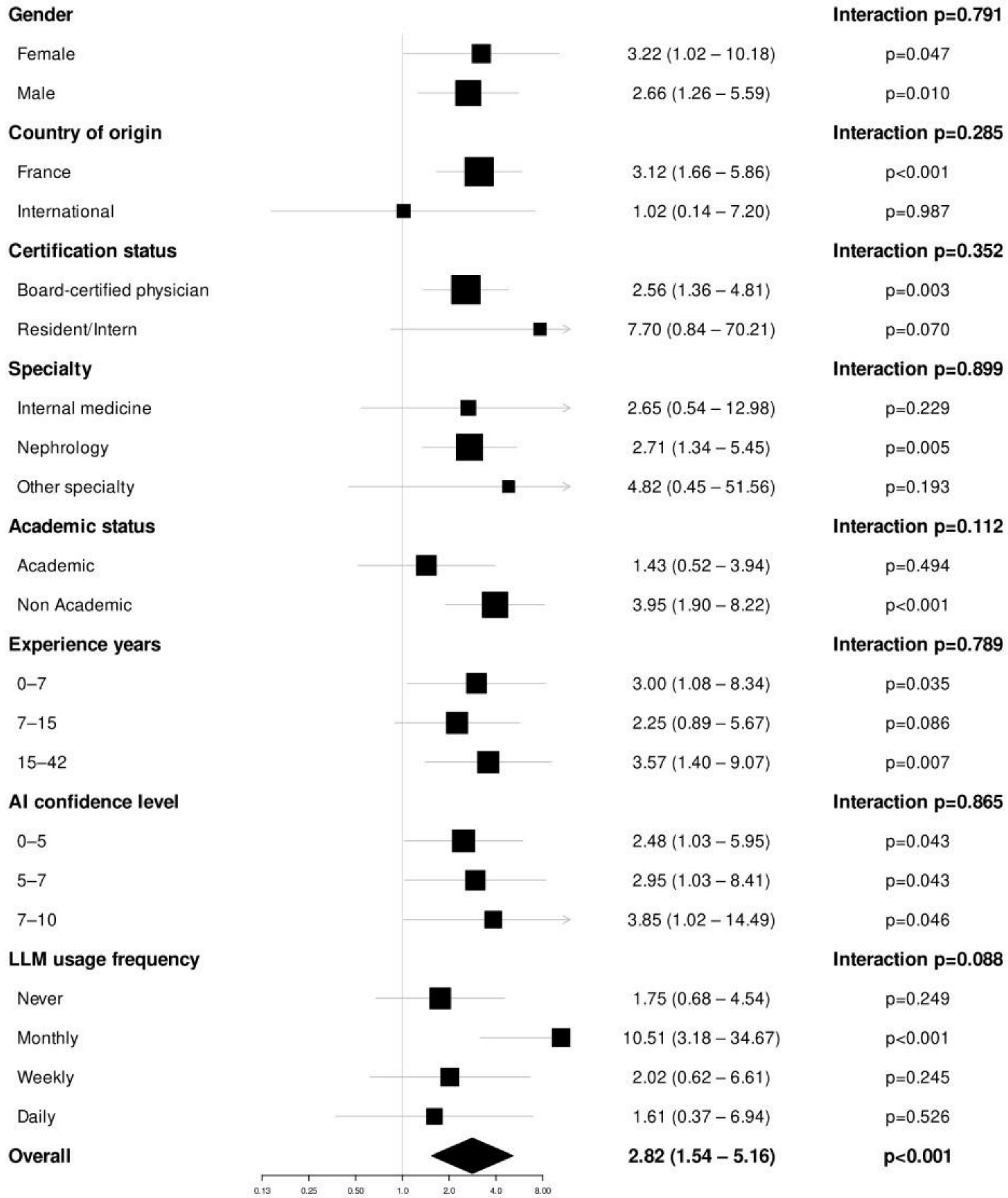

Subgroup-specific and overall adjusted odds ratios (with 95% confidence intervals) are shown for all prespecified subgroups. The overall estimate (OR 2.82, 95% CI 1.54–5.16; p<0.001) is displayed at the bottom. Interaction p values are reported for each subgroup variable.

Supplementary Figure S4. *Diagnostic revision patterns before and after AI suggestion (Sankey diagrams).*

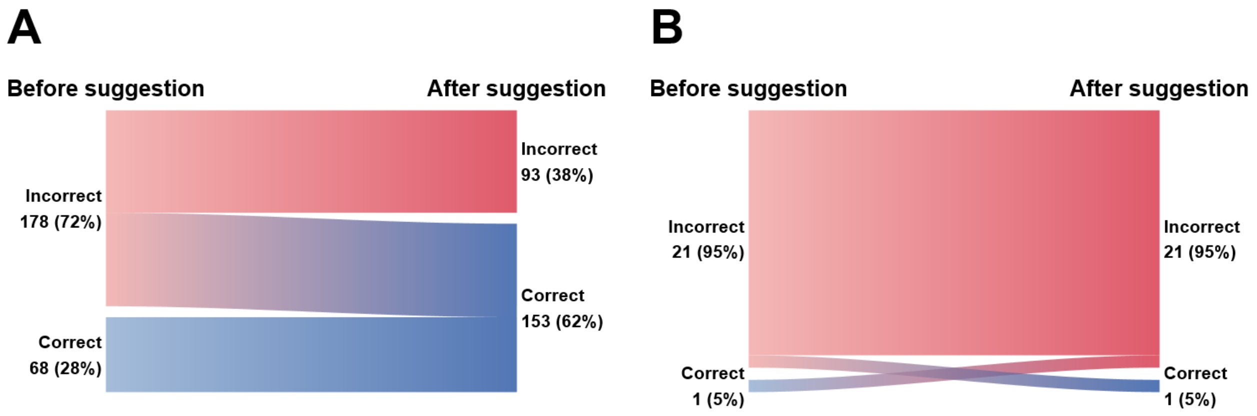

Panel A shows the flow of responses from before to after AI suggestion among all AI-arm evaluations (n=246), with proportions of correct and incorrect responses at each time point. Panel B shows the same flow restricted to responses that were initially correct (n=22), illustrating the rarity of AI-induced diagnostic errors.

Supplementary Figure S5. *Self-expressed diagnostic confidence across study groups.*

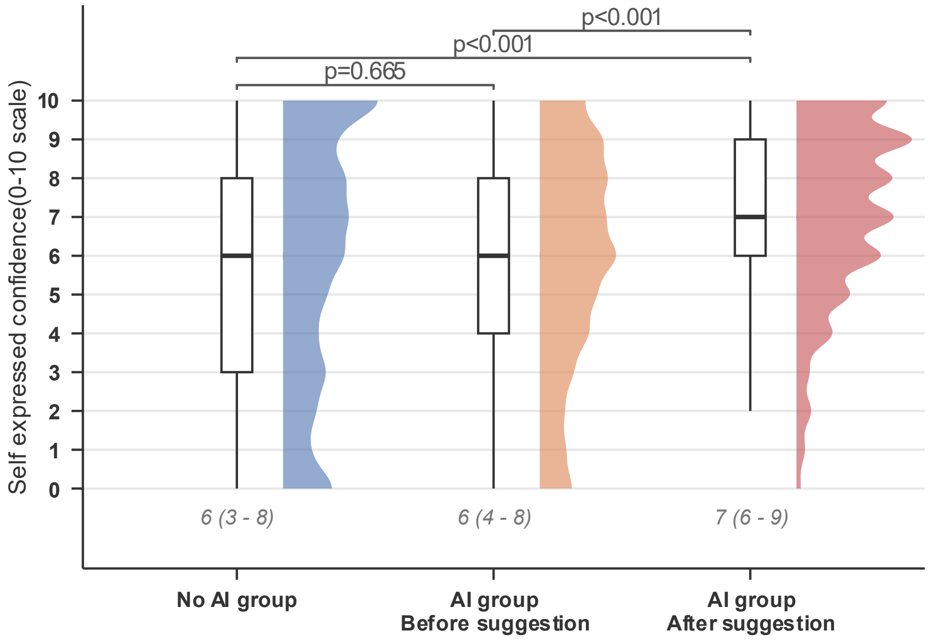

Raincloud plots show self-expressed diagnostic confidence (0–10 scale) in the No AI group, the AI group before suggestion, and the AI group after suggestion. Medians and interquartile ranges are indicated within each boxplot. Mixed-model p values are shown for pairwise comparisons (No AI vs. AI before: p=0.665; AI before vs. AI after: p<0.001; No AI vs. AI after: p<0.001).

TRIPOD-LLM Checklist

Section	Item	Description	Design	LLM task	Location
Title	1	Identify study as evaluating an LLM, specifying task, population, outcome.	All	All	Title
Abstract	2	See TRIPOD-LLM for abstracts.	All	All	Not applicable
Background	3a	Healthcare context and rationale for LLM evaluation.	All	All	Introduction
	3b	Target population and intended use of the LLM.	E, H		Introduction; Methods/Participants
Objectives	4	Study objectives including stage of evaluation.	All	All	Introduction (final para.)
Data	5a	Data sources for training, tuning, and/or evaluation.	All	All	Methods/Case Selection; Methods/AI Benchmarking
	5b	Data distribution and descriptors.	All	All	Results/Participants; Table 1
	5c	Date range of data used.	All	All	Methods/Case Selection
	5d	Data preprocessing and quality checking.	All	All	Methods/Case Selection
	5e	Handling of missing and imbalanced data.	All	All	Methods/Trial Procedures
Analytical methods	6a	LLM name, version, and last training date.	All	All	Methods/AI Benchmarking
	6b	LLM development process details.	M, D	All	Not applicable
	6c	Prompt engineering and inference settings.	M, D, E	All	Methods/AI Benchmarking
	6d	Initial and postprocessed LLM output.	All	All	Methods/Trial Procedures
	6e	Classification thresholds.	All	classification	Not applicable
LLM output	7a	Metrics capturing quality of generative outputs.	All	All	Results; Supp. Fig. S2
	7b	Outcome metrics' relevance to downstream task.	E, H	All	Methods/Trial Outcomes; Discussion
	7c	How LLM predictions were calculated and evaluated.	E, H	All	Methods
	7d	Qualifications of assessors and interrater agreement.	All	All	Methods/Trial Outcomes
	7e	Comparison to other LLMs and humans.	All	All	Results/Benchmarking; Supp. Fig. S2
Prompting	9a	Prompt design, curation, and selection.	All	All	Methods
	9b	Data used to develop prompts.	All	All	Methods
Compute	12	Compute or proxies (time, cost, machines).	M, D, E	All	Methods
Ethical approval	13	IRB name and informed consent.	All	All	Methods/Trial Design; Declarations
Open science	14a	Source of funding and role of funders.	All	All	Funding
	14b	Conflicts of interest.	All	All	Disclosure
	14c	Where protocol can be accessed.	H	All	Methods/Trial Design
	14d	Registration information.	H	All	Methods/Trial Design
	14e	Availability of study data.	All	All	Data Sharing Statement
	14f	Availability of analysis code.	All	All	Data Sharing Statement

Public involvement	15	Patient and public involvement.	H	All	Declarations
Participants	16a	Flow of participants through the study.	E, H	All	Results; Supp. Fig. S1
	16b	Characteristics by data source and splits.	E, H	All	Results; Table 1
	16d	Number of participants and outcome events.	E, H	All	Results/Primary Outcome
Performance	17	LLM performance per prespecified metrics.	All	All	Results; Supp. Figs. S3–S5
Interpretation	19a	Overall interpretation of main results.	All	All	Discussion
Limitations	19b	Limitations and generalizability.	All	All	Discussion
	19d	Intended use, end-user, and level of autonomy.	E, H	All	Introduction; Methods; Discussion
	19g	Next steps for future research.	All	All	Discussion

CONSORT 2025 Checklist

Section / Topic	No.	Checklist item	Location
Title and abstract	1a	Identification as a randomized trial	Title page
	1b	Structured summary of trial design, methods, results, conclusions	Not applicable
Open science	2	Trial registry name, number, and registration date	Methods/Trial Design
	3	Where protocol and statistical analysis plan can be accessed	Methods/Trial Design
	4	Data availability	Data Sharing Statement
	5a	Sources of funding and role of funders	Funding
	5b	Conflicts of interest	Disclosure
Background	6	Scientific background and rationale	Introduction
Objectives	7	Specific objectives related to benefits and harms	Introduction; Methods/Trial Outcomes
Patient and public involvement	8	Details of patient or public involvement	Declarations
Trial design	9	Type of trial, allocation ratio, and framework	Methods/Trial Design
	10	Important changes after trial commencement	Methods/Trial Design
Trial setting	11	Settings and locations	Methods/Trial Design; Results/Participants
Eligibility	12a	Eligibility criteria for participants	Methods/Participants
	12b	Eligibility criteria for sites or interventionists	Not applicable
Intervention	13	Intervention and comparator with sufficient detail for replication	Methods/Trial Procedures
Outcomes	14	Pre-specified primary and secondary outcomes	Methods/Trial Outcomes; Methods/Statistical Analysis
Harms	15	How harms were defined and assessed	Methods/Trial Outcomes; Results
Sample size	16a	How sample size was determined	Methods/Statistical Analysis
	16b	Interim analyses and stopping guidelines	Not applicable
Randomisation	17a	Who generated the allocation sequence and method	Methods/Participants
	17b	Type of randomisation and restrictions	Methods/Participants
Allocation concealment	18	Mechanism to implement the sequence	Methods/Participants
Implementation	19	Access to allocation sequence during enrolment	Methods/Participants
Blinding	20a	Who was blinded after assignment	Methods/Trial Design; Methods/Trial Outcomes
	20b	How blinding was achieved	Not applicable — open-label trial
Statistical methods	21a	Methods for comparing groups	Methods/Statistical Analysis
	21b	Who is included in each analysis	Methods/Statistical Analysis
	21c	How missing data were handled	Methods/Trial Procedures
	21d	Additional analyses (subgroup, sensitivity)	Methods/Statistical Analysis; Results
Participant flow	22a	Numbers randomized, receiving intervention, analyzed	Results/Participants; Supp. Fig. S1
	22b	Losses and exclusions after randomization	Supp. Fig. S1
Recruitment	23a	Dates of recruitment and follow-up	Methods/Trial Design
	23b	Why trial ended or was stopped	Not applicable — completed as planned
Intervention delivery	24a	Intervention as actually administered	Methods/Trial Procedures; Results/Participants
Baseline data	25	Baseline characteristics per group	Table 1 (main paper)
Outcomes	26	Results for each primary and secondary outcome	Results/Primary Outcome; Results/Secondary Outcomes
Harms	27	All harms or unintended events per group	Results; Discussion

Ancillary analyses	28	Subgroup and sensitivity analyses	Methods/Statistical Analysis; Results
Interpretation	29	Interpretation consistent with results	Discussion
Limitations	30	Trial limitations	Discussion
